# CBCT based investigation of frequency of Middle Mesial Canal in Mandibular First Molars of Saudi Sub-population

**DOI:** 10.12669/pjms.40.7.9101

**Published:** 2024-08

**Authors:** Ayman M. Abulhamael, Muhammad Qasim Javed, Sobia Hassan, Sundus Atique, Syed Rashid Habib

**Affiliations:** 1 Ayman M. Abulhamael Assistant Professor, Department of Endodontics, Faculty of Dentistry, King Abdulaziz University, Jeddah, Saudi Arabia; 2 Muhammad Qasim Javed Associate Professor, Department of Conservative Dental Sciences, College of Dentistry, Qassim University, Buraidah Qassim, Saudi Arabia; 3 Sobia Hassan Assistant Professor, Department of Periodontology, Islamic International Dental College and Hospital, Riphah International University, Islamabad 44000, Pakistan.; 4 Sundus Atique Research Assistant, College of Dental Medicine, Qatar University, QU Health, Doha Qatar; 5 Syed Rashid Habib Professor, Department of Prosthetic Dental Sciences, College of Dentistry, King Saud University, Riyadh 11545, KSA

**Keywords:** Cone beam computed tomography, Endodontics, Mandibular molars, Middle mesial canal

## Abstract

**Objective:**

One key factor in determining endodontic treatment outcome is the clinicians’ comprehension of tooth anatomy, as missed canals may harbor bacteria ultimately leading to apical periodontitis. The study aimed to investigate the prevalence of middle mesial canal (MMC) in Mandibular first molars (MFMs) of Saudi subpopulation.

**Methods:**

The cross-sectional retrospective study was conducted at Qassim University Dental College from June to August 2023. Overall, 302 CBCT images with 604 bilateral lower first molars were examined by two calibrated assessors. The existence of MMC was noted. The data were coded, and analysis was done in SPPS-24. The reliability of inter-evaluator and intra-evaluator agreement for detecting MMC were estimated using Cohen’s kappa.

**Results:**

The patients’ average age was 30.95±11.61years. The sample’s female to male ratio was 1:1.75. The overall frequency of MMC was 14.2%. The differences in the frequency of MMC on the basis of gender, quadrants and age groups were found to be insignificant. Inter-evaluator and intra-evaluator reliability was noted to be 0.78 and 0.74, depicting acceptable reliability.

**Conclusions:**

The MMC is an uncommon occurrence with rare bilateral presentation in MFMs of Saudi population. Endodontists performing endodontic treatment in such subjects should mindfully investigate inter mesiobuccal- mesiolingual canals area for locating, negotiating, and managing any present MMC’s.

## INTRODUCTION

Over the past decade, a heightened emphasis on restorative methods has been driven by a growing awareness of the benefits of preserving dental health and function. A healthy dentition not only serves its aesthetic role but is also important in maintaining the body’s overall health. It has been established in the literature that a good set of teeth, particularly the posterior teeth, is linked to a person’s nutritional state.^1^,^2^ Root canal treatment (RCT) is a procedure carried out for treatment of apical periodontitis (AP), inflammation of the periapical periodontium.^3^ RCT remains the primary approach in treatment of AP cases, allowing the retention of the patient’s affected tooth.^4^ The aim of performing RCT is to eliminate the endodontic microbial film and sealing the root canal system (RCS) in order to control the inflammatory reaction of periapical tissues.^5^

The success of RCT is dependent on a number of factors at every step of procedure. One key factor in determining the outcome of treatment is the clinician’s knowledge of tooth anatomy, as missed canals may harbour bacteria ultimately leading to post-operative or secondary AP.^6^,^7^ Within the human dentition, diverse anatomical variations have been documented for each type of tooth, including variations in both the number and shape of roots/root canals.^8^,^9^ If these variations go unnoticed, they can result in missed canals, causing these canals to evade the critical steps of disinfection and filling, ultimately leading to the failure of RCT.^10^ This highlights the importance of recognizing the variations in the anatomical configuration of RCS to ensure the success of RCT.

Mandibular first molar [MFM], the most common tooth to undergo RCT, usually exhibits two roots, and three canals (one canal in the distal root and two canals in the mesial root).^11^ However, like many other teeth, the MFM is susceptible to RCS variations. It may present with four canals, an extra distolingual root, two canals, a single canal or even a single root.^12^ One such variation is a third mesial canal; the middle mesial canal [MMC] which may be found in the mesial root.^11^ The presence of an extra mesial canal has been reported previously in a few studies, and has been given various titles; “accessory mesial canal”, “mesiocentral canal” and “middle mesial canal”.^13^ It has been suggested that the incidence of the MMC varies with ethnicity and can range anywhere from 10.8% to 27%.^13^

The use of cone beam computed tomography (CBCT) has recently gained popularity as a means to study the variations in canal and root morphology due to its accuracy.^14^ In contrast, traditional methods were limited to presenting structures in two-dimensional view, making them susceptible to distortion and superimposition.^15^ CBCT has been labelled as the ‘gold standard’ imaging technique for preoperative diagnosis in research studies and has been recognized for its sensitivity in detecting the MMC.^16^,^17^ Previous studies have found a correlation between the occurrence of MMC and ethnicity.^11^ Current study seeks to investigate the prevalence of MMC within Saudi subpopulation.

## METHODS

The cross-sectional retrospective study was conducted at Qassim University Dental College from June to August 2023 according to the recommendations for cross-sectional epidemiologic studies on root canal configuration and roots utilizing CBCT images.^18^ These images were obtained using Sirona CBCT Machine [Galileos Comfort: Beinshiem; Germany], with 160 μm voxel size, and 150X150 mm FOV. The analysis of CBCT scans was conducted on GALILEOS-viewer software (version 1.8.).

### Ethical Approval:

It was granted by the Scientific Research Deanship, Qassim University (Approval no: 21-05-01, Dated: 15/12/21).

Scalex sample size (SS) calculator was utilized for SS calculation with 95% confidence interval, 7% expected prevalence and 5% precision.^11^,^19^ A minimum SS calculated was 101 CBCT scans containing 202 bilateral MFMs. The CBCT scans obtained from Qassim University Dental Clinics were assessed for data extraction, confirming with the guidelines noted in the position statement of American association of Endodontists.^16^ Collected demographic details included patients’ age and gender. CBCT conducted from 2020 to 2023 for various reasons, including maxillo-facial trauma, treatment planning for dental implants, orthodontic management, and bilateral MFMs were assessed for the research. Whereas, the CBCT images with missing MFMs, MFM’s with immature roots or periapical pathology were not included.

The outcome (Prevalence of MMC) was evaluated by two evaluators (endodontists with > 12 years’ experience) who underwent calibration by utilizing 10 CBCT images. To evaluate the reliability of their assessments, their inter-evaluator and intra-evaluator agreement for detecting MMC were estimated using Cohen’s kappa based on the analysis of 10 CBCT images. The scans assessment involved a 3D evaluation of MFMs mesial root in coronal, sagittal, and axial dimensions. This assessment was conducted after aligning the long axis of the roots with reference lines provided by visualization software. Both evaluators were allowed to make adjustments of various tools, and visualization settings, including filters, and noise reduction to improve image quality. The presence of MMC was noted as yes or no. Additionally the gender and age of the patients was also recorded.

### Statistical Analysis:

The data was analyzed using SPPS version 24.0. Descriptive variables were recorded as frequencies and percentages. To assess the association between the prevalence of MMC and both gender and jaw side, a chi-square test was employed.

## RESULTS

**Fig.1:**
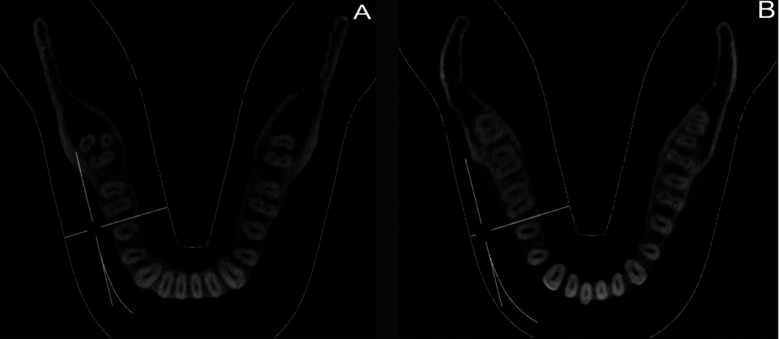
(A) Bilateral Middle Mesial Canal in teeth number 36 and 46 (B) Unilateral Middle Mesial Canal in tooth number 36.

**Fig.2:**
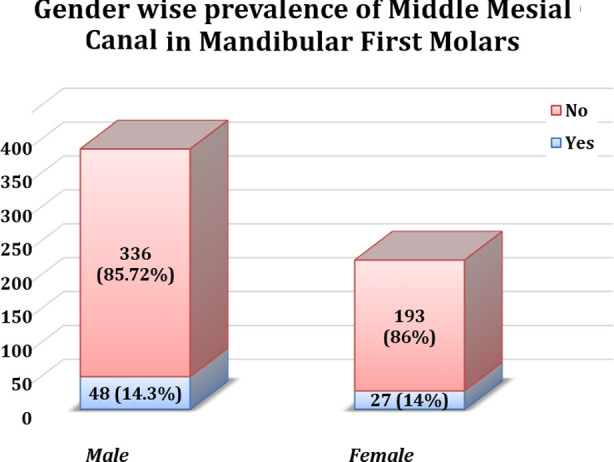
Gender wise prevalence of Middle Mesial Canal in Mandibular First Molars.

Total 543 CBCT images were screened in current study. A total of 241 scans were not included in final analysis, with single (109) and Bilateral (131) missing MFMs. One scan was excluded as it had artifacts. The final analysis was carried out for 604 bilateral MFM in 302 CBCT scans. The sample’s female to male ratio was 1:1.75, 63.6% males (n=192) and 36.4% females (n=110). The mean age of the patients was 30.95±11.61 ranging between 11 and 63 years. The overall frequency of MMC was 14.2%. The differences in the frequency of MMC on the basis of gender, quadrants (Fig.1) and age groups were found to be insignificant (Fig.2, Table-I and Table-II). Table-III is depicting the analysis of unilateral/bilateral presence of MMC according to gender. Inter-evaluator and intra-evaluator reliability was noted to be 0.78 and 0.74, depicting acceptable reliability.

**Table-I:** Prevalence of Middle Mesial Canal in Mandibular First Molars by Quadrant.

		Middle Mesial Canal N(%)			P-Value*

		No	Yes	Total	
Tooth Type	Mandibular Left First Molar	263 (85.2)	39 (14.8)	302 (50)	0.71
	Mandibular Right First Molar	266 (86.5)	36 (13.5)	302 (50)	
Total		529 (85.8)	75 (14.2)	604 (100)	

* Chi-Square.

**Table-II:** Age group wise prevalence of Middle Mesial Canal in Mandibular First Molar.

		Middle Mesial Canal N(%)			P-Value*

		No	Yes	Total	
Age Group	11-30	308	42	350 (57.9)	0.9
	31-50	185	27	212 (35.1)	
	51-70	36	6	42 (6.95)	
Total		529 (85.8)	75 (14.2)	604 (100)	

* Chi-Square.

**Table-III:** Middle Mesial canal prevalence in mandibular first molars by gender and jaw quadrant (n = 604).

Gender	Quadrant Side	No of Patients	No of Teeth	Middle Mesial Canal
Male	Bilateral	10	20	20 (3.79%)
	Unilateral	28	28	28 (5.31%)
	Total	38	48	48 (9.1%)
Female	Bilateral	6	12	12 (2.26%)
	Unilateral	15	15	15 (2.83%)
	Total	21	27	27 (5.1%)
Grand Total		53	75	75 (14.2%)

## DISCUSSION

Numerous studies have been conducted to investigate the variations in the morphology of MFMs.^10^,^11^,^20^ The need for effective RCT, involving thorough canal disinfection, rests upon accurately identifying the pulp chamber and canals. Understanding anatomical variations plays a crucial role in ensuring the provision of appropriate treatment. Consequently, several studies have identified potential indicators that may signal the existence of the MMC, including an increased distance between mesio-lingual and mesio-buccal canals or the presence of an isthmus between the two canals. 13

In our study, the overall prevalence of the MMC was determined to be 14.2%, which falls within the incidence range reported in a multinational survey, ranging from 1% to 23%.^11^ In a comparable study conducted by Bhatti et al in Pakistan, a comparable incidence of MMC was observed in the sample population, specifically 14.7%.^13^ This prevalence was greater than that observed in Thai (0.22%) and Chinese (1.79%) populations. However, it was notably less than prevalence found in Egyptian population (27.5%).^21^-^23^ It has been theorized that MMC occurrence might be more frequent within specific ethnic or racial groups, and this population variation could account for the discrepancies observed in these studies’ findings.^11^ Our study found insignificant results when the prevalence of MMC was compared between males and females. These findings are concurrent with other studies.^11^,^13^,^24^,^25^ However, other studies have suggested a significant difference in MMC prevalence amongst genders.^26^,^27^ Furthermore, the overall frequency of MMC reported in current study was higher than frequency documented by Aldosimani et al (1.4%) and Mashyakhy et al (4.2%) in other studies on Saudi population. 25,28

It has been proposed that age not only plays a significant role in MMC prevalence, but also in its configuration. Although our study did not find any significant differences in the percentages among different age groups, previous research has reported a significant association between age and incidence of MMC.^24^,^29^ Peiris et al. documented an increase in inter-canal communications up to age of 20-40 years, after which this number started to decrease. It was noted that up to age of 11, the mesial canal of the MFM typically appears as a single structure, and approximately 3-6 years later, the process of canal differentiation begins.^30^ The deposition of secondary dentine islands has been proposed as a plausible cause for this separation of canals.^31^ As individuals continue to age beyond 40 years, there may be a complete disappearance or narrowing of these canals.^32^ This phenomenon could potentially account for the variations observed in different studies.

Our findings revealed statistically insignificant difference in the prevalence of MMC when considering the quadrant of the jaw, suggesting that the likelihood of encountering MMCs was fairly uniform across all quadrants. Similar results were presented in other studies.^11^,^13^,^25^,^33^ Various in vivo and ex-vivo techniques have been used to study the morphological differences in teeth.^24^ Among the in vivo approaches commonly employed to study canal configurations are guided troughing and CBCT.^34^ Conventional access cavity preparations may limit the identification of MMCs due to small orifice diameters.^35^ Guided troughing under magnification and CBCT serve as suitable alternatives to the conventional approach for the detection of these canals. Several studies have utilized guided troughing, suggesting that magnification under dental operating microscope and troughing combined may significantly enhance MMC identification.^36^

Our study used CBCT for the identification of MMC. The use of CBCT for assessing the complexity of the RCS has been endorsed in recent years, providing a non-invasive three-dimensional view of intricate anatomy.^10^,^11^,^13^,^14^ A study conducted by Matherne et al revealed that 41% of endodontists failed to identify at least one canal when periapical radiographs were used in comparison to CBCT.^37^ However, it is essential to consider the potential radiation exposure associated with CBCT. Exposure to radiation should only be justified when the overall diagnostic benefits significantly outweigh any potential harm or uncertainty for the patient’s safety.^38^

The study provides insight into the prevalence of the MMCs in MFMs among the Saudi population. The utilization of CBCT scans allowed a comprehensive and precise assessment of RCS. These findings highlight the importance of thorough clinical examination and imaging techniques in the diagnosis and treatment planning for RCT, emphasizing the need for dental practitioners to remain vigilant to the diverse anatomical variations that may be encountered in their patients.

### Limitations of study:

It is important to acknowledge certain limitations in the study that warrant consideration. Firstly, the study did not account for potential variations in the morphology of the canals associated with MMCs. By not accounting for these morphological variations, the study may have overlooked important morphological factors that could influence MMC prevalence. Additionally, while CBCT scans are a reliable tool for MMC detection, they are also subject to artefacts. These artefacts can potentially introduce errors in the data, affecting the accuracy of MMC detection and prevalence calculations.

### Strengths of study:

The study possesses notable strengths. Firstly, it is contributing towards the scientific data on MMC prevalence. Moreover, the study has reasonable sample size, which can provide a valuable guidance for drawing conclusions about MMC prevalence in the population under study. Lastly, the utilization of CBCT scans offered a significant advantage as it allows non-invasive and objective means of detecting MMCs which minimizes the potential for observer bias and subjectivity in the results, ensuring that the data collected accurately reflects the presence or absence of MMCs.

## CONCLUSIONS

The MMC is an uncommon occurrence with rare bilateral presentation in MFMs of Saudi population. Endodontists performing RCT in such subjects should mindfully investigate inter mesiobuccal- mesiolingual canals area for locating, negotiating, and managing any present MMC’s.

### Future Recommendations:

Future study with larger sample size and the utilization of Micro-CT for the detection of MMC is recommended that will contribute towards the enhancement of clinicians understanding of the anatomical variations and the endodontic treatment outcomes.

### Authors’ Contribution:

**AMA:** Conceived, did the data collection and write up- editing, and review of manuscript.

**MQJ:** Conceived, designed, did the data collection, statistical analysis, write up & final approval of manuscript and project supervision.

**SH:** Statistical analysis and write up- original draft preparation.

**SA:** Statistical analysis and write up- editing, and review of manuscript.

**SRH:** Designed, project administration and write up- original draft preparation.

**MQJ:** Is responsible and accountable for the accuracy or integrity of the work.
